# Empirical nexus between financial inclusion and carbon emissions: Evidence from heterogeneous financial economies and regions

**DOI:** 10.1016/j.heliyon.2023.e13164

**Published:** 2023-02-28

**Authors:** Shahzad Hussain, Raazia Gul, Sabeeh Ullah, Abdul Waheed, Muhammad Naeem

**Affiliations:** a Department of Business Administration, Rawalpindi Women University, Pakistan; b Faculty of Management Sciences, Shaheed Zulfikar Ali Bhutto Institute of Science & Technology, Karachi, Pakistan; c IBMS, Faculty of Management and Computer Sciences, The University of Agriculture Peshawar, Pakistan; d Faculty of Management Sciences, Foundation University Islamabad, Pakistan

**Keywords:** Financial inclusion, Sustainable environment, Carbon emissions, Heterogeneous financial economies

## Abstract

We aim to investigate the empirical nexus between carbon emissions and financial inclusion for a panel of 74 countries from 2004 to 2020 based on the environment kuznets curve (EKC). Using the advanced panel data analysis framework of Driscoll–Kraay, Generalised linear model, and Prais-Winsten test for the entire sample and heterogeneous subsamples, we document an inverted U-shape relationship between carbon emissions and inclusive financial system. Notably, an inverted U-shape relationship is established in developed, emerging and frontier economies except in standalone economies. Furthermore, the analysis of region-wise subsamples reveals that nonlinear relationship varies across regions. The heterogeneous response of financial inclusion in curtailing environmental degradation provides vital policy insights. It suggests that financial inclusion can be used as a mitigation measure based on well-structured and robust regulatory and legal frameworks. These frameworks would create synergy effects of financial inclusion in designing policies and addressing issues related to sustainable development and climate change.

## 1 Introduction

The augmented awareness of climate change and environmental sustainability calls for low-carbon economies. The significance of reduced carbon emissions (CO2) has been more pronounced after recent initiatives such as 26th United Nations Climate Change Conference (COP26),^1^ Paris Climate Change Act,^2^ Energy Bill,^3^ among others. The United Nations' sustainable development goals (SDGs)^4^ urge countries to take serious actions to combat climate change and its impacts by reducing carbon emissions. During the COP26, participants agreed that money is a vital tool to achieve the said goals. However, the transition to carbon neutrality and environmental sustainability is impossible without improving financial services, resources and markets [1]. Nevertheless, financial and economic development should be well aligned with SDGs as otherwise, it may add to carbon emissions and environmental degradation [2,3].

In the economic development process, the role of financial inclusion has received massive attention and interest from policymakers and researchers [4]. Besides the role of financial inclusion in technological progress and sustainable economic growth, contemporary researchers argue that an inclusive financial system has equally affected the quality of the environment, particularly carbon emissions [5]. Theoretically, an inconclusive relationship exists between an inclusive financial system and environmental sustainability [[6], [7], [8]].

One strand of researchers argued that financial inclusion enhances environmental sustainability and reduces CO2 emissions through increased spending on research & development and technological innovations [9,10]. More specifically, governments and firms tend to adopt environmentally friendly technologies that reduce carbon emissions and enhance environmental sustainability due to the low cost of borrowing [11]. On the contrary, other strands of literature argue that an accessible financial system with affordable financing boosts industrial and manufacturing activities, increasing CO2 emissions and damaging environmental quality [12,13].

Besides this, the extant literature also supports the nonlinear relationship between carbon emissions and financial sector development [8,14]. Ref. [10] find an asymmetric impact of financial inclusion on CO2 that varies geographically. Moreover, environmental degradation is vast if the country is in an early development phase. In contrast, economic growth enhances ecological preservation in the later development phase. Empirically, Refs. [15,16] find an inverted U-shaped relationship between an inclusive financial system and CO2 emission. Likewise, Ref. [8] observe a nonlinear relationship between carbon emission and financial sector development. However, they argued that the nonlinearity between environmental quality and an inclusive financial system is linked to a robust governance mechanism. Therefore, strict adherence to environmental regulations based on environment, social, and governance (ESG) is the key to ensuring sustainable development. In other words, this relationship may not be nonlinear in those countries or regions with weak ESG-related governance mechanisms. Hence, the nonlinear nexus between environmental sustainability and financial inclusion may vary across different levels of economic development and governance mechanisms.

These inconsistencies in the empirical literature create an opportunity to revisit the empirical nexus between an inclusive financial system and CO2 emissions. The objective of our study is to investigate the link between CO2 and financial inclusion, preserving the heterogeneity of financial development of sample economies and regions.

Our study offers various contributions to the literature. *First*, contemporary empirical studies by Refs. [8,17] have failed to consider different phases of the financial development of countries while investigating the empirical nexus between the inclusive financial system and CO2 emissions. As technological progress, economic growth and capital accumulation could vary across countries. Countries with well-established financial systems may have less information asymmetry and low-cost funds available to households and businesses compared to poorly developed financial systems [18]. However, it remains unclear from the prior literature whether different phases of financial development matter when investigating the nonlinear nexus between an inclusive financial system and CO2 emissions. Keeping this in view, the current study investigates the empirical nexus between CO2 emissions and inclusive financial system in heterogeneous financial economies such as developed, developing, frontier, and standalone economies. *Second*, our study aids in fully comprehending the carbon emissions – financial inclusion nexus to fight climate change and to make a close estimate of CO2 emissions across various regions. It is accomplished by testing financial inclusion-based EKC in multiple areas. If diverse EKC situations are discovered for different emission types, a one-size-fits-all strategy cannot be the best course of action. As discussed above, it is crucial to closely evaluate the impact of inclusive financial systems on carbon emissions across various regions to give policymakers more significant insights.

We test the Environmental Kuznets Curve (EKC) hypothesis to identify the association between CO2 emissions and inclusive financial development across different regions. These regions, Africa, America, Asia, Europe and the Middle East, are of great importance to be investigated for various reasons. *First*, these regions broadly vary along levels of financial, economic and governance [[19], [20], [21]]. *Second*, these regions have experienced rapid economic development and globalisation over the last few years. However, such a pronounced economic development is subject to environmental consequences [1,22,23]. For instance, the Asia region recently produced 17.74 billion metric tons of carbon emissions in 2021, which is higher than the total emissions in all regions in 2021.

Furthermore, North America is the next most polluting region in the same year, with 5.6 billion metric tons of carbon emissions. Overall, carbon emissions in most of these regions increased by 5% in 2021 as compared to 2020 levels (Tiseo, 2022).^5^ *Third*, the scant empirical literature provides insufficient support to the EKC hypothesis testing region-wise comparisons of financial inclusion – carbon emissions nexus.

The rest of the paper is structured as follows: a brief review of the literature and hypothesis development are presented in section 2, followed by material and methods in section 3. Results based on empirical analysis are discussed in section 4, followed by a conclusion in section 5.

## 2 Literature review

### 2.1 Theoretical perspective of financial inclusion and environmental degradation

Financial development cannot be separated from financial inclusion. Therefore, discussing financial development’s empirical and theoretical underpinnings is vital to building a connection between an inclusive financial system and CO2 emissions [1,24]. Theoretically, researchers have differing opinions about how related inclusive financial development and environmental sustainability are (Jiang & Ma, 2019; Zaidi et al., 2021) [25,26]. One constituent of the literature suggests that financial development lowers CO2 emissions by mitigating financial constraints with low borrowing costs for upgrading equipment and production technologies [27]. Likewise, Ref. [28] suggest that financial growth promotes the reduction of carbon emissions.

On the other hand, the extant literature contends that increased carbon emissions caused by financial development worsen environmental degradation. Because a well-functioning financial system provides low-cost financing for expanding production capacity, which leads to deterioration of environmental quality [29]. The third strand of literature supports nonlinearity between CO2 emissions and inclusive financial progress [14]. Theoretically, Ref. [29] introduced the concept of EKC. They argued that environmental quality is linked with the economic development phases: a low development stage is associated with environmental degradation, whereas the advantages of strong economic development preserve the environment quality.

### 2.2 Empirical literature and hypothesis development

The literature has paid huge attention to the empirical nexus between CO2 emissions and inclusive financial system in recent years [4]. However, the results are conflicting; few studies have found a negative relationship between an inclusive financial system and CO2 emissions, while others have documented a positive association between them. Studies that support that financial progress lowers environmental quality include [1,4,[30], [31], [32],^33^] among others. The existing literature recognises the constructive role of financial development in struggling with inclusive economic growth, climate change and sustainable development [34]. For instance, Ref. [31] investigated data from 39 SSA countries between 2004 and 2014 and found that economic growth in SSA countries lowers carbon emissions. According to Ref. [1], who used a static panel data framework, there is an unfavourable link between CO2 emissions and financial development in China.

The reduction of carbon emissions and other pollutants is positively correlated with an inclusive financial system, according to Shahbaz et al. (2022) [10], who studied 30 Chinese provinces from 2011 to 2017. The same idea is supported for China by Ref. [33] using an ARDL approach. Ref. [32] also classified 33 OECD economies into two groups based on their degree of globalisation: highly globalised and lower globalised. The study found that the impact of inclusive financial progress on CO2 emission was negative for each group. While analysing a panel dataset of 19 developing countries from 1990 to 2013, Ref. [35] confirmed the negative influence of financial progress on CO2 and argued that financial progress contributes to environmental sustainability. Ref. [1] consider an advanced panel data framework for 23 economies from 1985 to 2011 and support that financial growth reduces CO2 emissions.

On the contrary, many studies claim that financial inclusion deteriorates environmental quality [7,25,[36], [37], [38], [39]]. Using Driscoll-Kraay standard errors panel data models for 31 Asian countries from 2004 to 2014, Ref. [7] find that inclusive financial growth led to higher CO2 emissions in the sample region. Similarly, through second-generation panel data techniques, Ref. [37] documented that financial inclusion increased CO2 emissions. Additionally, Ref. [40] looked into the long-term and short-term relationships between inclusive financial and CO2 emissions from 1971 to 2010 using the Panel ARDL technique. Their research shows that financial inclusion causes the environment to degrade much more. Ref. [41] found that inclusive financial significantly and positively influence CO2 emissions in South Asia.

The literature also supports the nonlinear relationship between financial growth and environmental sustainability. For instance, Ref. [42] determine the nonlinearity between CO2 emissions and inclusive financial for Central Asian and European economies from 2010 to 2019 and confirm the inverted U-Shaped EKC. While taking 25 OECD economies from 1971 to 2007, Ref. [43] found a nonlinear relationship between CO2 emissions and financial growth. Ref. [8] used the EKC framework and suggested an inverted U-shaped relationship was found between CO2 emissions and financial development. Similarly, Ref. [44] confirms the inverted U-shaped relationship between CO2 emissions and financial growth of 52 countries from 2001 to 2014. Using the Panel Dynamic Common Correlated Effects (CCE) approach, Ref. [17] examined the OIC countries from 2004 to 2018. Their findings support the nonlinear relationship between inclusive financial development and CO2 emissions for all OIC countries, including all panels. The literature review is summarised in Table 1. Based on the literature above, we hypothesise as follows:

H1 There exists an inverted U-shaped EKC relationship between financial CO2 emissions and financial inclusion.

Notably, the extant literature advances the literary work and offers valuable insights, but the empirical nexus between inclusive financial and CO2 emissions are inconclusive and inconsistent. The two gaps primarily support further deliberation and exploration of this nexus. *First,* although there is a sufficient amount of research on the FI-CO2 emissions nexus, the existing literature is still at its infancy stage when it comes to the said nexus regarding the level of financial development of countries. Our study examines the EKC hypothesis between financial inclusion and carbon emissions in heterogeneous financial economies such as developed, frontier, developing and standalone capital markets. While analysing the effects of inclusive financial on CO2 emissions, Ref. [8] neglected to consider the different phases of financial inclusion in sample countries. Hence, the study argues that financial inclusion for facilitating economic growth, capital accumulation, and technological progress differs through governments with different phases of financial development. *Second*, the study tests the EKC hypothesis between the inclusive financial system and CO2 emissions across different regions such as America, Asia, Europe, the Middle East and Africa through advanced statistical techniques such as Driscoll–Kraay standard errors (D-K), GLM (Generalised Linear Model), and Prais-Winsten to produce consistent and efficient parameters as a robustness check since these regions vary along economic development, political, and level of governance. Our study is unique in providing fresh evidence about the heterogeneous and nonlinear relationship between carbon emissions and financial inclusion.

**Table 1:** Summary of empirical studies.

**No**	Authors	Sample Countries	Sample Period	Objectives	Findings
**1**	Odhiambo (2020)	39 sub-Saharan African (SSA)	2004–2014	To examine the inclusive financial development-CO2 emissions nexus.	FI↑ CO 2 ↓
**2**	Zhao and Yang (2020)	China	2001–2015	To identify the impact of financial development on carbon emissions?	FD↑ CO 2 ↓
**3**	Gokmenoglu and Sadeghieh (2019)	Turkey	1960–2011	To investigate the nexus between financial system development, CO2 emissions and energy consumption in the long run.	FD↑ CO 2 ↓ FUEL CON ↑ CO 2 ↑
**4**	Le et al. (2020)	Asia (31 Countries)	2004–2014	To examine the linkages between financial inclusion and CO2 emissions.	FI ↑ C02↑
**5**	Kayani et al. (2020)	10 Countries	1990–2016	To investigate the association between CO2 emission and financial inclusion.	FD↑ C02↑
**6**	Jiang and Ma (2019)	155 Countries	1990–2014	This study examine the relationship between financial development and carbon emissions	FD ↑CO 2 ↑
**7**	Chunyu et al. (2021)	European and central Asian economies	2010–2019	To investigate the nonlinear inclusive financial-carbon emissions nexus	Nonlinear relationship (Supported)
**8**	Imran et al. (2022)	OIC countries	2004–2018	To examine the inverted U-shaped relationship of financial inclusion with CO2 emissions	Nonlinear relationship (Supported)
**9**	Renzhi and Baek (2020)	103 Countries	2004–2014	To examine the nonlinear relationship between financial inclusion and carbon emission	Inverted U shaped relationship was found between FI and C02 (EKC exist)
**10**	Hung et al. (2018)	25 OECD countries	1971–2007	To investigate the impact of development of financial system on CO2 emissions.	FD inverted U-Shape CO 2(Supported)
**11**	Shahbaz et al. (2022)	China	2011–2017	To explore whether FI has an impact on collaborative reduction of CO2 and pollutant emissions in China	The impact of financial inclusion on CO2 emissions is asymmetric and heterogeneous.
**12**	Zaidi et al. (2021)	OECD countries	2004–2017	To examine the dynamic linkages between financial inclusion, energy consumption and carbon emissions	FI ↑ C02↑
**13**	Qin et al. (2021)	E7 countries	2004–2016	To explore the quantile relationship between FI and CO2 emissions.	FI↑ CO 2 ↓
**14**	Akinsola et al. (2022)	Brazil	1983–2017	To assess the effect of public-private partnerships in energy and financial development on Brazil's ecological environment	Economic growth leads to environmental degradation
**15**	Qin et al. (2021a)	China	1988–2018	To identify the role of FD along with renewable energy, output, human capital and financial risk index on carbon emissions.	An improvement in FD causes to reduce carbon emissions in China.
**16**	Liu et al. (2022)	Asian Countries	1995–2019	To examines the impact of FI on the environment-economic performance	FI ↑ C02↑
**17**	Jinpeng et al. (2022)	South Asian Countries	1980–2019	To examine the association between FI and carbon emissions through a quantile technique.	Nonlinear relationship was found between FI and carbon emissions

## 3 Research methodology

### 3.1 Research design

The data related to financial inclusion, carbon emissions, and macroeconomic indicators were collected from Global Findex Database and World Development Indicators (WDI). For this purpose, a sample of 74 heterogeneous financial economies consisting of developed markets (22), developing markets (22), Frontier Markets (19), and Standalone Markets (11) is based on MSCI classification. For further analysis, these sampled countries were also divided into different region such as Europe, the Middle East and Africa regions (50), Asia (13) and America (11). The sample details are provided in Appendix Table A1.

### 3.2 Operationalisation of variables

For empirical analysis, the study categorises the variables into a dependent variable (carbon emissions), an independent variable (financial inclusion). The study also employed a few control variables, which include the gross domestic product (GDP), trade openness (TO), population growth (POG), industrialisation (IND), and human capital (HC).

In literature, the concept of financial inclusion has recently emerged. We constructed financial inclusion index based on five different proxies. These proxies are (i) bank deposit amount (ii) bank credits, (iii) number of commercial bank branches available for 100,000 adults, (iv) number of ATMs available for 100,000 adults, and (v) Institutions of commercial banks [7,45]. The first two represent usage, and the latter three indicate financial services' availability [46]. By following [47], all these proxies were normalised using the following normalisation techniques via equations [[1], [2], [3], [4]]:

a) Z-score normalisation: The z-score normalisation will be measured as follows.

(1) $$Z-score=\frac{X_{i}-\bar{X}}{\delta }$$

where, $\bar{X}$ is a group average and δ is a standard deviation.

b) min-max normalisation: The minimum and maximum observations are used to measure normalised scores, as under.

(2) $$min-\max=\frac{X_{i}-X_{\min}}{X_{\max}-X_{\min}}$$

where, $X_{\min}$ and $X_{\max}$ are the minimum and maximum values in the data set respectively.

c) Softmax normalisation: Using exponential functions, mean and standard deviation, it calculates normalised scores, as follows.

(3) $$softmax=\frac{1}{1+e^{-\left(\frac{X-\bar{X}}{\delta }\right)}}$$

where, e is the exponent, $\bar{X}$ is a group average and δ is a standard deviation.

Principal component analysis (PCA) was used to construct an index for financial inclusion. As, prior literature most widely used PCA due to several reasons. First, to lessen the dimensionality of data and build composite indicators index, PCA removes excess information and extracts hidden relationships and features [48]. Second, we used two tests before the PCA, namely Bartlett's and Kaiser-Meyer-Olkin (KMO) to confirm the validity of the chosen proxies to develop an index for financial inclusion. The value of Bartlett's test was significant (P < 0.05) and the value of KMO (>0.5) indicates the suitability of PCA. The results are reported in Table 2.

**Table 2:** Results of Bartlett and KMO tests.

	Bartlett test of sphericity			Kaiser-Meyer
Full Sample				
FI	1139.108***	6.0000	0.0000	0.7150
Developed Markets				
FI	122.630***	6.0000	0.0000	0.7460
Emerging Markets				
FI	320.425***	6.0000	0.0000	0.5490
FI	476.429***	6.0000	0.0000	0.6690
Standalone Markets				
FI	234.228***	6.0000	0.0000	0.5290
Europe, Middle East & Africa				
FI	693.261***	6.0000	0.0000	0.6730
Asia				
FI	409.424***	6.0000	0.0000	0.7600
Americas				
FI	296.721***	6.0000	0.0000	0.7510

PCA is performed in two steps. (i) various components were estimated to identify the lowest pairwise correlation and the variations in the original variable. (ii) only those components were retained whose eigen values was greater than 1 to build the Financial inclusion index (Gujarati & Porter, 2009). Table 3, Table 4 report the cumulative variations of each component and pattern matrix of PCA for whole sample as well as subsamples.

**Table 3:** Total variance explained and Pattern Matrix (Sub-Sample).

Total variance explained			
Full Sample			
Component	Eigenvalue	Proportion	Cumulative
Comp1	2.33668	0.5842	0.5842
Comp2	0.788853	0.1972	0.7814
Comp3	0.471182	0.1178	0.8992
Comp4	0.403286	0.1008	1
Developed Markets			
Comp1	1.63028	0.4076	0.4076
Comp2	1.07342	0.2684	0.6759
Comp3	0.839009	0.2098	0.8857
Comp4	0.457292	0.1143	1
Emerging Markets			
Comp1	1.76535	0.4413	0.4413
Comp2	1.36809	0.342	0.7834
Comp3	0.587542	0.1469	0.9302
Comp4	0.279015	0.0698	1
Frontier Markets			
Comp1	2.56218	0.6405	0.6405
Comp2	0.898357	0.2246	0.8651
Comp3	0.306533	0.0766	0.9418
Comp4	0.232925	0.0582	1
Standalone Markets			
Comp1	2.21768	0.5544	0.5544
Comp2	1.08312	0.2708	0.8252
Comp3	0.513759	0.1284	0.9536
Comp4	0.185448	0.0464	1
Europe, Middle East & Africa			
Comp1	2.21074	0.5527	0.5527
Comp2	0.89968	0.2249	0.7776
Comp3	0.463803	0.116	0.8936
Comp4	0.425777	0.1064	1
Asia			
Comp1	2.86239	0.7156	0.7156
Comp2	0.491131	0.1228	0.8384
Comp3	0.447267	0.1118	0.9502
Comp4	0.199207	0.0498	1
Americas			
Comp1	2.86239	0.7156	0.7156
Comp2	0.491131	0.1228	0.8384
Comp3	0.447267	0.1118	0.9502
Comp4	0.199207	0.0498	1

**Table 4:** PCA pattern matrix.

Full Sample				
Variable	PC1	PC2	PC3	PC4
FI-1	0.4913	0.4979	0.7118	0.0638
FI-2	0.4961	0.4781	−0.6952	0.2049
FI-3	0.5358	−0.3275	−0.0713	−0.775
FI-4	0.4748	−0.6452	0.0703	0.5944
Developed Markets				
Variable	PC1	PC2	PC3	PC4
FI-1	0.6272	0.193	−0.3909	−0.6454
FI-2	0.582	−0.4221	−0.3055	0.6243
FI-3	0.2615	0.8572	0.1596	0.4139
FI-4	0.4467	−0.2229	0.8535	−0.1495
Emerging Markets				
Variable	PC1	PC2	PC3	PC4
FI-1	0.1539	0.714	0.6507	0.2078
FI-2	0.3584	0.6027	−0.6601	−0.2694
FI-3	0.666	−0.2274	−0.131	0.6982
FI-4	0.6358	−0.2744	0.3517	−0.6299
Frontier Markets				
Variable	PC1	PC2	PC3	PC4
FI-1	0.5368	−0.3091	−0.6638	0.4192
FI-2	0.4527	−0.6251	0.6349	−0.0352
FI-3	0.5538	0.2761	−0.1657	−0.7679
FI-4	0.4475	0.6614	0.359	0.4831
Standalone Markets				
Variable	PC1	PC2	PC3	PC4
FI-1	0.4896	0.3719	−0.7869	0.053
FI-2	0.411	0.6351	0.5766	0.3085
FI-3	0.607	−0.2366	0.2169	−0.727
FI-4	0.4721	−0.6343	0.0351	0.6112
Europe, Middle East & Africa				
Variable	PC1	PC2	PC3	PC4
FI-1	0.5336	−0.3204	0.6759	−0.3948
FI-2	0.476	−0.5914	−0.3276	0.5625
FI-3	0.5421	0.2753	−0.6007	−0.5191
FI-4	0.4414	0.6869	0.2739	0.5082
Asia				
Variable	PC1	PC2	PC3	PC4
FI-1	0.4789	−0.7119	−0.2649	0.4401
FI-2	0.5453	−0.0351	−0.2484	−0.7998
FI-3	0.4774	0.6994	−0.3476	0.4027
FI-4	0.4954	0.053	0.8645	0.067
Americas				
Variable	PC1	PC2	PC3	PC4
FI-1	0.4797	0.3954	0.7575	0.1995
FI-2	0.4817	0.613	0.5428	0.3123
FI-3	0.511	0.5862	0.1436	0.6121
FI-4	0.5261	0.3525	0.3332	0.6986

### 3.3 Econometric model

For theoretical foundation, the influence of inclusive financial system on CO2 emissions is investigated using the stochastic effects by regression on population, affluence, and technology (STIRPAT) [49]. The STIRPAT model is:

(4) $$I_{it}=\gamma_{0}+\gamma_{1}P_{it}+\gamma_{2}A_{it}+\gamma_{3}T_{it}+\varepsilon_{it}$$

where I_it,_ P_it,_ A_it_, and T_it_ are environmental effects, population, affluence and technology for country i at time t, respectively. For financial inclusion, we extend the STIRPAT model. Several control variables, namely gross domestic product (GDP), trade openness (TO), population growth (POG), industrialisation (IND), and human capital (HC) are added based on the previous literature [8,50] Z. Khan et al., 202. The baseline model is:

(5) $${Co2}_{it}=\delta_{0}+\delta_{1}{FI}_{it}+\delta_{2}{FI}_{it}^{2}+\delta_{3}{GDP}_{it}+\delta_{4}{GDP}_{it}^{2}+\sum_{j=1}^{n}\gamma_{j}{Controls}_{it}+\varepsilon_{it}$$

where ${Co2}_{it}$ is the logarithm of CO2 emissions, ${FI}_{it}$ is the financial inclusion index, ${FI}^{2}$ is the squared term of Financial inclusion (An inverted U-shaped EKC curve when $\delta_{1}$ > 0, $\delta_{2}$ <0). GDP is the gross domestic product and ${GDP}^{2}$ stands for the squared term of GDP. Further, Control represents the control variables such as trade openness, industrialisation, population growth, and human capital.

### 3.4 Estimation techniques

The panel data was analysed using the following steps to empirically examine the impact of inclusive financial on CO2 emissions across different regions: (i) Cross-sectional dependency was checked using the cross-sectional dependence test proposed by Ref. [51]. (ii) For Eq. (5), we employed Driscoll–Kraay (D-K) standard errors in the full sample. Driscoll–Kraay is appropriate in case of cross-sectional dependency and short time period [45]. Moreover, Driscoll–Kraay deals with the problem of heteroscedasticity, serial correlation and contemporaneous correlation both in balanced and unbalanced panels [52]. (iii) Using Driscoll–Kraay procedure, the empirical nexus between inclusive financial and CO2 emissions was also analysed in subsamples of heterogeneous financial economies and across different regions. (iv) For robustness check, the study also employed GLM (Generalised linear model), and Prais-Winsten test in the entire sample and subsamples.

## 4 Results and discussions

### 4.1 Summary statistics

**Fig. 1:**
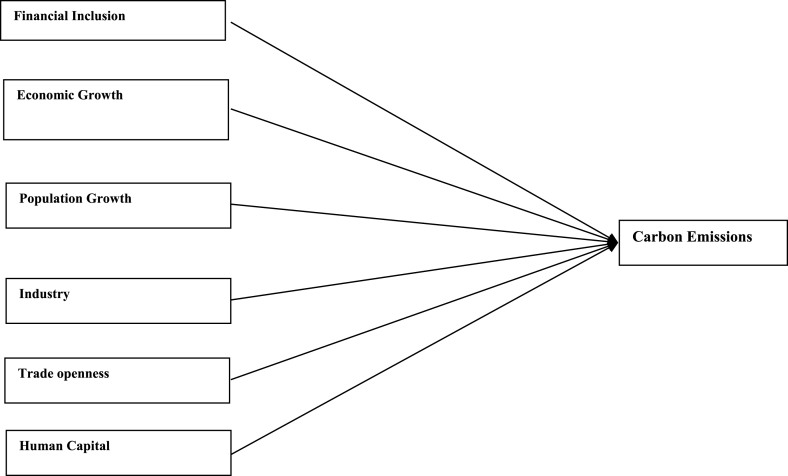
Conceptual Framework of the study.

The findings of summary statistics for each variable are shown in Table 5. The carbon emission averages and standard deviations (M = 7.374, SD = 6.341) are somewhat lower than the whole sample values given by Ref. [8]. The summary statistics show that the average number of ATMs and branches of commercial banks per 100,000 adults are higher than those reported in Ref. [8]. Similarly, the outstanding deposits and loans (per cent GDP) is also higher than reported by Ref. [7] for the Asia region. This shows that certain countries have better financial inclusiveness than others. The mean and standard deviation of trade openness are among the control variables (M = 90.435, SD = 55.717). Furthermore, Fig. 1 shows that the relationship between carbon emission and financial inclusion is nonlinear and makes an inverted U-shaped relationship over the sample period. Moreover, carbon emissions kept increasing even when financial inclusion decreased from 2009 to 2013 with small dips. Although since 2008, both financial inclusion and carbon emissions started growing, the rate of increase for carbon emissions was relatively high compared to that of financial inclusion. Both carbon emissions and financial inclusion declined abruptly in 2020, on the onset of Covid-19.^6^ The nonlinear trend of financial inclusion and carbon emissions in subsamples such as developed, emerging, and frontier markets. However, Fig. 5 reveals the linear trend of financial inclusion and CO2 emissions in standalone countries.

**Table 5:** Description of variables.

	Symbol	Description	Obs	Mean	Std. Dev.	Min	Max
Carbon Emission	CO2	CO2 in MC/Capita	1258	7.374	6.341	0.209	47.7
Financial inclusion (Proxy 02)	FI-1	Number of ATM machines available for 100,000 adults	1258	65.121	44.534	0.129	228.417
Financial inclusion (Proxy 01)	FI-2	Number of commercial banks' branches for 100,000 adults	1258	22.146	16.359	0.41	104.34
Financial inclusion (Proxy 04)	FI-3	Banks' Outstanding loans divided by GDP	1258	71.00	40.873	0.186	304.575
Financial inclusion (Proxy 03)	FI-4	Banks' Outstanding deposits divided by GDP	1258	64.241	38.8	8.572	342.116
Population	POG	Population Growth	1258	1.173	1.861	−2.258	17.512
Economic Growth	GDP	GDP per capita	1258	26.061	1.69	22.208	30.696
Industry	IND	Industry value added divided by GDP	1258	28.409	10.436	7.241	74.812
Trade openness	TO	The sum of imports and exports divided by GDP	1258	90.435	55.717	20.723	437.327
Human Capital	HC	The returns to education	1258	2.919	0.522	1.578	4.352

### 4.2 Cross-sectional dependence tests

The results of cross-sectional dependency test using [51] are reported in Table 6. Ref. [7] documented that CD test are best suited to shorter time series but larger cross-sectional data. The results of CD tests for the full sample and subsamples reveal that all the variables are statistically significant at 1% level, rejecting the null hypothesis of cross-sectional independence. Hence, cross-sectional dependency exists amongst the variable across full sample and subsamples.

**Table 6:** Results from Cross-section independence tests.

Panel A: Development based Classification										
	Full Sample		Developed		Emerging		Developing		Standalone	
CO2	2.39***	0.000	9.64***	0.000	2.73***	0.000	7.96***	0.000	6.21***	0.000
FI	2.60***	0.000	5.23***	0.000	21.84***	0.000	9.65***	0.000	1.93***	0.000
GDP	156.93***	0.000	41.60***	0.000	47.62***	0.000	42.52***	0.000	23.57***	0.000
POPG	2.35***	0.000	2.09***	0.000	1.87*	0.062	3.323***	0.000	4.21***	0.000
IND	84.93***	0.000	34.90***	0.000	29.46***	0.000	19.71***	0.000	5.02***	0.000
TO	31.75***	0.000	19.61***	0.000	4.52***	0.000	8.17***	0.000	2.74***	0.000
HC	7.60***	0.000	12.981***	0.000	2.26***	0.000	2.50***	0.000	8.76***	0.000
Panel B: Region Based Classification										
		Europe, Middle East & Africa								
CO2		3.01***	0.0000			Asia			Americas	
FI		4.81***	0.0000			5.01***	0.0000		5.24***	0.000
GDP		102.54***	0.0000			9.92***	0.0000		7.78***	0.000
POPG		9.444***	0.0000			30.97***	0.0000		26.45***	0.03
IND		55.31***	0.0000			3.6***	0.0000		2.17**	0.000
TO		33.94***	0.0000			13.82***	0.0000		12.69***	0.000
HC		1.87**	0.0610			10.75***	0.0000		4.26***	0.000
						2.18***	0.0000		8.30***	0.000

### 4.3 Financial inclusion and carbon emissions (full and subsamples)

We analysed the baseline Eq. (5) through advanced statistical techniques in the full sample and subsamples to achieve consistent and efficient estimates even in serial correlation, heteroscedasticity and cross-sectional dependency. Table 7 reports the results of the nonlinear effect of financial inclusion on carbon emissions for the full sample. The financial inclusion coefficient is significant and positive. In contrast, the coefficient of a square of financial inclusion was found to be negative (δ1 > 0, δ2<0), indicating an inverted U-shaped relationship between CO2 and inclusive financial. Our results support the EKC hypothesis for the whole sample in the long run. Additionally, our findings are consistent with the theory that, in the early stages, a sound financial system provides a mechanism for mitigating risk and effective resource pooling that promotes industrial activities for better economic prospects though worsening environmental quality. Later on, after a turning point, economic development ensures environmental preservation [8,42].

**Table 7:** The Nonlinear relationship between Financial Inclusion and Carbon Emissions (Full Sample).

	D-K			GLM			Prais-Winsten		
Z_FI	0.292***			0.292***			0.313***		
	(0.0154)			(0.0168)			(0.0315)		
Z_FI2	−0.0602***			−0.0602***			−0.0601***		
	(0.00222)			(0.00654)			(0.0105)		
Soft_FI		0.265***			0.265***			0.284***	
		(0.0127)			(0.0152)			(0.0290)	
Soft_FI2		−0.0596***			−0.0596***			−0.0687***	
		(0.00192)			(0.00694)			(0.0122)	
mmx_FI			0.290***			0.290***			0.226***
			(0.0163)			(0.0164)			(0.0273)
mmx_FI2			−0.0621***			−0.0621***			−0.0450***
			(0.00243)			(0.00671)			(0.00899)
Popg	0.0334**	0.0317**	0.0320**	0.0334***	0.0317**	0.0320**	−0.00561	−0.00570	−0.00518
	(0.0145)	(0.0139)	(0.0149)	(0.0129)	(0.0129)	(0.0128)	(0.00569)	(0.00569)	(0.00571)
l_gdp	0.0989	0.0878	0.177***	0.0989	0.0878	0.177**	0.127*	0.132*	0.201***
	(0.0596)	(0.0587)	(0.0250)	(0.0761)	(0.0758)	(0.0772)	(0.0745)	(0.0744)	(0.0759)
l_gdp2	−0.114	−0.0905	0.354***	−0.114	−0.0905	0.354***	0.322**	0.337**	0.498***
	(0.110)	(0.109)	(0.0568)	(0.130)	(0.130)	(0.131)	(0.137)	(0.137)	(0.139)
IND	0.0403***	0.0403***	0.0413***	0.0403***	0.0403***	0.0413***	0.0218***	0.0220***	0.0201***
	(0.00182)	(0.00183)	(0.00161)	(0.00219)	(0.00221)	(0.00218)	(0.00362)	(0.00363)	(0.00372)
TO	0.000872**	0.000748**	0.000970***	0.000872**	0.000748**	0.000970***	0.00224***	0.00211***	0.00310***
	(0.000314)	(0.000324)	(0.000275)	(0.000373)	(0.000372)	(0.000368)	(0.000670)	(0.000669)	(0.000670)
hc	0.857***	0.865***	0.859***	0.857***	0.865***	0.859***	0.122***	0.128***	0.131***
	(0.0339)	(0.0322)	(0.0339)	(0.0431)	(0.0427)	(0.0427)	(0.0424)	(0.0423)	(0.0426)
Constant	−4.635***	−4.351**	2.513***	−4.635**	−4.351**	2.513	3.849**	3.992**	5.678***
	(1.521)	(1.498)	(0.663)	(1.996)	(1.991)	(2.036)	(1.938)	(1.936)	(1.976)
F-Stats (P Value)	0.000	0.000	0.000				0.000	0.000	0.000
Time Fixed Effects	Yes	Yes	Yes	Yes	Yes	Yes	Yes	Yes	Yes
R-squared	0.696	0.698	0.699				0.489	0.494	0.463
Observations	1258	1258	1258	1258	1258	1258	1258	1258	1258
No of Countries	74	74	74	74	74	74	74	74	74

**Fig. 2:**
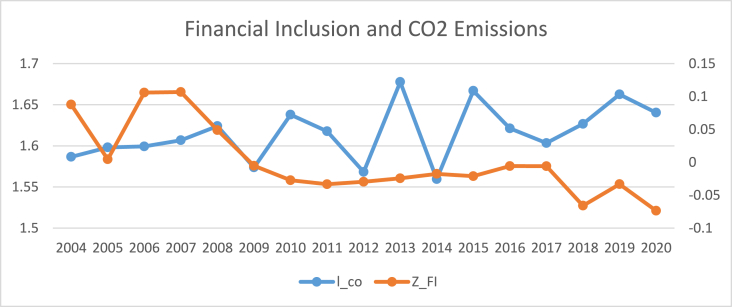
The annual cross sectional averages of financial inclusion (FI) and log of carbon emission (co) for full sample.

**Fig. 3:**
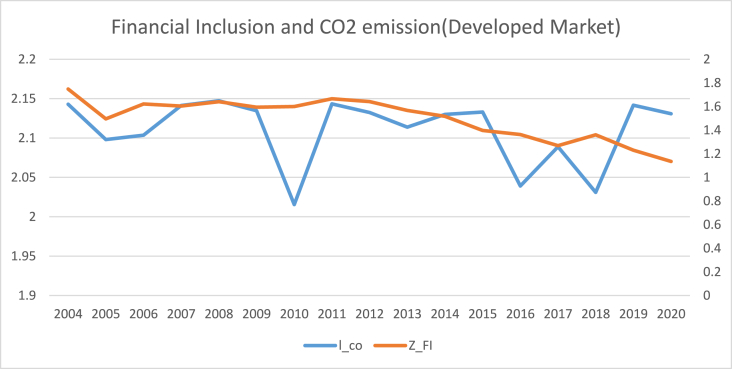
The annual cross-sectional averages of financial inclusion (FI) and log of carbon emission (co) for Developed Countries.

**Fig. 4:**
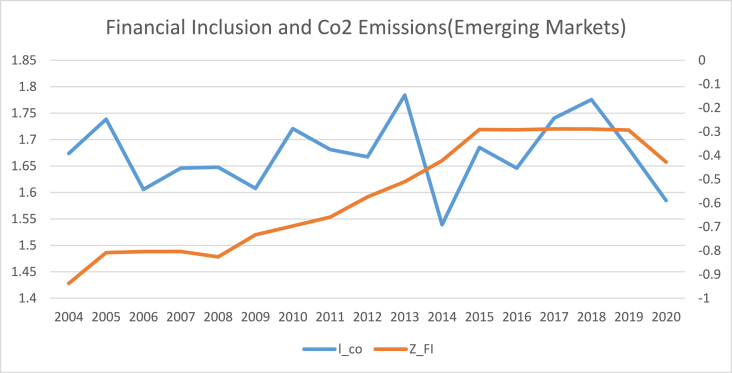
The annual cross-sectional averages of financial inclusion (FI) and log of carbon emission (co) for Emerging Countries.

**Fig. 5:**
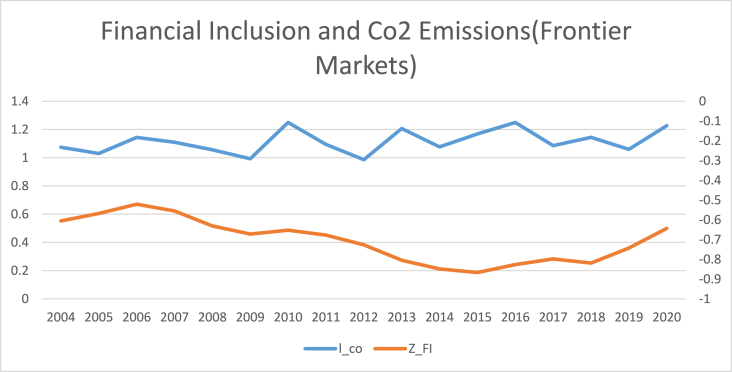
The annual cross-sectional averages of financial inclusion (FI) and log of carbon emission (co) for Frontier Countries.

**Fig. 6:**
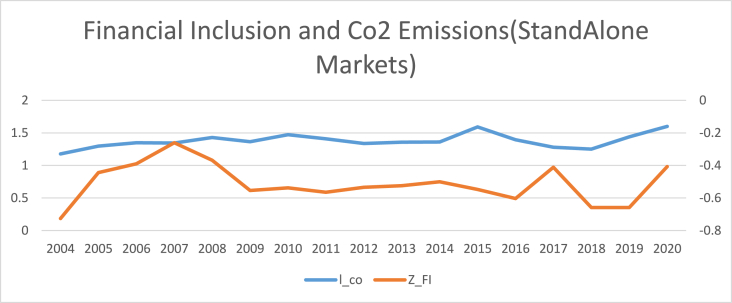
The annual cross-sectional averages of financial inclusion (FI) and log of carbon emission (co) for Standalone Countries.

Table 8, Table 9, Table 10, Table 11 reports the results of the nexus between inclusive financial development and CO2 emissions for developed, emerging, frontier and standalone capital markets. The coefficients of financial inclusion and squared term of financial inclusion reveal that the EKC hypothesis exists in developed, emerging and frontier economies. However, the EKC hypothesis does not hold in standalone economies. Our findings align with the theoretical assumptions that different countries may affect financial development regarding green economic growth, technical advancement, and capital accumulation. For instance, compared to the countries with less developed financial systems, those with well-established financial systems have high technological progress, economic expansion, reduced information asymmetry, and lower costs for people and businesses to obtain credit [4]. As a result, it is unlikely to expect that nations at different stages of financial development would see the same effect on carbon emissions from inclusive financial progress [43]. The graphical representation of the nexus between financial inclusion and carbon emissions in Fig. 2, Fig. 3, Fig. 4, Fig. 5 also confirms that an inverted U-shaped relationship exists in developed, emerging, frontier and standalone capital markets except for standalone economies. Likewise, Fig. 6 also confirms that linear nexus of financial inclusion and CO2 emission in standalone economies.

**Table 8:** The Nonlinear relationship between Financial Inclusion and Carbon Emissions (Developed Markets).

	D-K			GLM			Prais-Winsten		
Z_FI	0.0990*			0.0990**			0.0838***		
	(0.0557)			(0.0482)			(0.0401)		
Z_FI2	−0.0269**			−0.0269**			−0.0293***		
	(0.0137)			(0.0121)			(0.00193)		
Soft_FI		0.145*			0.145**			0.132***	
		(0.0872)			(0.0573)			(0.0350)	
Soft_FI2		−0.0412*			−0.0412***			−0.0453*	
		(0.0243)			(0.0157)			(0.0253)	
mmx_FI			0.138***			0.138***			0.1864*
			(0.0465)			(0.0468)			(0.0558)
mmx_FI2			−0.0374***			−0.0374***			−0.0263*
			(0.0128)			(0.0119)			(0.0139)
popg	0.0566***	0.0528***	0.0549**	0.0566*	0.0528*	0.0549*	0.00823	0.00750	0.00957
	(0.0191)	(0.0177)	(0.0199)	(0.0290)	(0.0290)	(0.0286)	(0.0172)	(0.0172)	(0.0171)
l_gdp	0.0321	0.0462	0.000110	0.0321	0.0462	0.000110	0.0280	0.0292	0.0447
	(0.0424)	(0.0485)	(0.0305)	(0.0896)	(0.0899)	(0.0883)	(0.107)	(0.107)	(0.106)
l_gdp2	0.0459	0.0115	0.0917*	0.0459	0.0115	0.0917	0.132	0.125	0.153
	(0.0495)	(0.0576)	(0.0452)	(0.146)	(0.147)	(0.141)	(0.177)	(0.178)	(0.175)
IND	0.0124***	0.0139***	0.0142***	0.0124**	0.0139***	0.0142***	0.0159**	0.0171**	0.0168**
	(0.00230)	(0.00310)	(0.00226)	(0.00527)	(0.00536)	(0.00521)	(0.00803)	(0.00814)	(0.00796)
TO	0.000135	0.00003	0.0001	0.000135	.0000375	0.0001	0.0007	0.00009	.0000252
	(0.000170)	(0.000209)	(0.00016)	(0.0003)	(0.000304)	(0.00029)	(0.000533)	(0.0005)	(0.00053)
hc	0.304***	0.315***	0.306***	0.304***	0.315***	0.306***	0.114*	0.117*	0.119*
	(0.0675)	(0.0659)	(0.0675)	(0.0675)	(0.0669)	(0.0669)	(0.0659)	(0.0657)	(0.0657)
Constant	−0.231	−0.676	0.553	−0.231	−0.676	0.553	1.969	1.954	2.356
	(1.124)	(1.293)	(0.825)	(2.374)	(2.390)	(2.315)	(2.836)	(2.835)	(2.817)
R-squared	0.230	0.236	0.244				0.781	0.782	0.782
F-Stats (P Value)	0.000	0.000	0.000				0.000	0.000	0.000
Time Fixed Effects	Yes	Yes	Yes	Yes	Yes	Yes	Yes	Yes	Yes
Observations	374.00	374.00	374.00	374.00	374.00	374.00	374.00	374.00	374.00
No of Countries	22.00	22.00	22.00	22.00	22.00	22.00	22.00	22.00	22.00

**Table 9:** The Nonlinear relationship between Financial Inclusion and Carbon Emissions (Emerging Markets).

	D-K			GLM			Prais-Winsten		
Z_FI	0.224***			0.224***			0.261***		
	(0.0375)			(0.0481)			(0.0693)		
Z_FI2	−0.275***			−0.275***			−0.0830*		
	(0.0268)			(0.0403)			(0.0423)		
Soft_FI		0.237***			0.237***			0.226***	
		(0.0301)			(0.0408)			(0.0616)	
Soft_FI2		−0.199***			−0.199***			−0.0729**	
		(0.0306)			(0.0313)			(0.0339)	
mmx_FI			0.278***			0.278***			0.173***
			(0.0462)			(0.0451)			(0.0602)
mmx_FI2			−0.189***			−0.189***			−0.173***
			(0.0319)			(0.0342)			(0.0316)
popg	0.0577***	0.0576***	0.0615***	0.0577***	0.0576***	0.0615***	−0.00816	−0.00828	−0.00833
	(0.00939)	(0.0111)	(0.0118)	(0.0186)	(0.0188)	(0.0190)	(0.00622)	(0.00623)	(0.00608)
l_gdp	0.0273	0.0205	0.530***	0.0273	0.0205	0.530***	0.159	−0.156	0.187
	(0.0995)	(0.0978)	(0.160)	(0.163)	(0.164)	(0.184)	(0.137)	(0.137)	(0.140)
l_gdp2	−0.0112	−0.0163	0.911***	−0.0112	−0.0163	0.911***	0.0804	0.0823	0.173
	(0.138)	(0.136)	(0.164)	(0.267)	(0.269)	(0.296)	(0.287)	(0.288)	(0.299)
IND	0.0427***	0.0428***	0.0434***	0.0427***	0.0428***	0.0434***	0.0238***	0.0238***	0.0188***
	(0.00210)	(0.00231)	(0.00214)	(0.00375)	(0.00379)	(0.00385)	(0.00505)	(0.00511)	(0.00525)
TO	0.000134	6.46e-05	0.000608	0.000134	6.46e-05	0.000608	0.00230	0.00223	0.00346*
	(0.00135)	(0.00132)	(0.00108)	(0.00117)	(0.00118)	(0.00119)	(0.00180)	(0.00181)	(0.00184)
hc	0.767***	0.773***	0.805***	0.767***	0.773***	0.805***	0.114	0.115	0.109
	(0.165)	(0.181)	(0.162)	(0.100)	(0.101)	(0.102)	(0.0748)	(0.0749)	(0.0739)
Constant	−2.381	−2.235	11.96**	−2.381	−2.235	11.96**	4.770	4.695	5.466
	(2.887)	(2.877)	(4.645)	(4.320)	(4.349)	(4.899)	(3.543)	(3.547)	(3.605)
R-squared	0.648	0.642	0.631				0.494	0.491	0.438
F-Stats (P Value)	0.000	0.000	0.000				0.000	0.000	0.000
Time Fixed Effects	Yes	Yes	Yes	Yes	Yes	Yes	Yes	Yes	Yes
Observations	374.00	374.00	374.00	374.00	374.00	374.00	374.00	374.00	374.00
No of Countries	22.00	22.00	22.00	22.00	22.00	22.00	22.00	22.00	22.00

**Table 10:** The Nonlinear relationship between Financial Inclusion and Carbon Emissions (Frontier Markets).

	D-K			GLM			Prais-Winsten		
Z_FI	0.316***			0.316***			0.343***		
	(0.0382)			(0.0408)			(0.0743)		
Z_FI2	−0.0334***			−0.0334**			−0.0384**		
	(0.00709)			(0.0160)			(0.0188)		
Soft_FI		0.289***			0.289***			0.296***	
		(0.0343)			(0.0379)			(0.0702)	
Soft_FI2		−0.0251**			−0.0251***			−0.0687**	
		(0.0112)			(0.0103)			(0.0289)	
mmx_FI			0.319***			0.319***			0.261***
			(0.0360)			(0.0400)			(0.0657)
mmx_FI2			−0.0422***			−0.0422**			−0.0670**
			(0.0110)			(0.0210)			(0.0260)
popg	0.0165	0.0111	0.0188	0.0165	0.0111	0.0188	0.00937	0.00898	0.0146
	(0.0423)	(0.0416)	(0.0425)	(0.0335)	(0.0336)	(0.0336)	(0.0206)	(0.0204)	(0.0206)
l_gdp	0.00726	0.0347	0.356***	0.00726	0.0347	0.356**	0.117	0.114	0.284*
	(0.0825)	(0.0801)	(0.0852)	(0.161)	(0.159)	(0.169)	(0.151)	(0.151)	(0.155)
l_gdp2	0.411	0.458	1.012***	0.411	0.458	1.012***	0.319	0.344	0.548
	(0.276)	(0.263)	(0.284)	(0.335)	(0.332)	(0.356)	(0.369)	(0.372)	(0.383)
IND	0.0388***	0.0394***	0.0414***	0.0388***	0.0394***	0.0414***	0.00780	0.00609	0.00866
	(0.00998)	(0.0106)	(0.0109)	(0.0109)	(0.0111)	(0.0109)	(0.0111)	(0.0111)	(0.0111)
TO	0.00859***	0.00844***	0.00830***	0.00859***	0.00844***	0.00830***	0.00531**	0.00511**	0.00685***
	(0.00119)	(0.00120)	(0.00128)	(0.00189)	(0.00191)	(0.00190)	(0.00210)	(0.00211)	(0.00210)
hc	0.867***	0.888***	0.858***	0.867***	0.888***	0.858***	0.164**	0.169**	0.150*
	(0.0676)	(0.0666)	(0.0756)	(0.0927)	(0.0933)	(0.0927)	(0.0809)	(0.0805)	(0.0817)
Constant	−2.344	−1.706	6.750**	−2.344	−1.706	6.750	3.341	3.391	7.521*
	(2.185)	(2.093)	(2.429)	(4.245)	(4.208)	(4.512)	(3.932)	(3.937)	(4.073)
Observations	224	224	224	224	224	224	224	224	224
R-squared	0.792	0.793	0.796				0.361	0.365	0.371
F-Stats (P Value)	0.000	0.000	0.000				0.000	0.000	0.000
Time Fixed Effects	Yes	Yes	Yes	Yes	Yes	Yes	Yes	Yes	Yes
Observations	323.00	323.00	323.00	323.00	323.00	323.00	323.00	323.00	323.00
No of Countries	19.00	19.00	19.00	19.00	19.00	19.00	19.00	19.00	19.00

**Table 11:** The Nonlinear relationship between Financial Inclusion and Carbon Emissions (Standalone Markets).

	D-K			GLM			Prais-Winsten		
Z_FI	0.0703			0.0703			0.124		
	(0.0462)			(0.0472)			(0.0803)		
Z_FI2	−0.0434**			−0.0434*			−0.0826**		
	(0.0151)			(0.0256)			(0.0412)		
Soft_FI		0.0370			0.0370			0.0386	
		(0.0655)			(0.0483)			(0.0856)	
Soft_FI2		−0.0410*			−0.0410*			−0.0951**	
		(0.0204)			(0.0247)			(0.0413)	
mmx_FI			0.0615*			0.0615			0.0988
			(0.0352)			(0.0409)			(0.0663)
mmx_FI2			−0.0545***			−0.0545**			−0.0743**
			(0.0148)			(0.0241)			(0.0363)
Popg	−0.111**	−0.113***	−0.123***	−0.111***	−0.113***	−0.123***	−0.0409	−0.0392	−0.0549
	(0.0399)	(0.0377)	(0.0354)	(0.0386)	(0.0384)	(0.0387)	(0.0405)	(0.0404)	(0.0411)
l_gdp	0.340***	0.347***	0.454***	0.340**	0.347**	0.454***	0.290	0.311	0.393*
	(0.0471)	(0.0450)	(0.0726)	(0.141)	(0.143)	(0.148)	(0.207)	(0.207)	(0.206)
l_gdp2	0.885***	0.906***	1.123***	0.885***	0.906***	1.123***	1.017**	1.063***	1.215***
	(0.0983)	(0.0975)	(0.141)	(0.258)	(0.261)	(0.271)	(0.388)	(0.388)	(0.380)
IND	0.00105	0.00162	0.00355	0.00105	0.00162	0.00355	−0.00135	−0.000772	0.00299
	(0.00289)	(0.00270)	(0.00345)	(0.00748)	(0.00753)	(0.00719)	(0.0105)	(0.0104)	(0.0101)
TO	0.00251***	0.00309**	0.00304***	0.00251**	0.00309**	0.00304***	0.00349**	0.00472***	0.00413***
	(0.000699)	(0.00118)	(0.000791)	(0.00113)	(0.00125)	(0.00112)	(0.00149)	(0.00169)	(0.00138)
Hc	0.694***	0.711***	0.598***	0.694***	0.711***	0.598**	−0.200	−0.172	−0.127
	(0.106)	(0.120)	(0.122)	(0.252)	(0.254)	(0.257)	(0.215)	(0.215)	(0.219)
Constant	8.337***	8.402***	11.54***	8.337**	8.402**	11.54***	9.883*	10.22*	12.16**
	(1.287)	(1.228)	(1.977)	(3.816)	(3.875)	(4.055)	(5.550)	(5.558)	(5.556)
Observations	106	106	106	106	106	106	106	106	106
R-squared	0.701	0.696	0.707				0.529	0.528	0.538
F-Stats (P Value)	0.000	0.000	0.000				0.000	0.000	0.000
Time Fixed Effects	Yes	Yes	Yes	Yes	Yes	Yes	Yes	Yes	Yes
Observations	187.000	187.000	187.000	187.000	187.000	187.000	187.000	187.000	187.000
No of Countries	11.000	11.000	11.000	11.000	11.000	11.000	11.000	11.000	11.000

In a nutshell, the inclusive financial system and its square terms have a significant positive and negative impact, respectively on the intensity of carbon emission. Similarly, several studies, such as [42,53] supported the notion that the non-monotonic impact of financial inclusion is not always linear on CO2 emissions. As a result, the link between inclusive financial systems and carbon emission intensity is inverted U-shaped in developed, emerging and frontier economies. Consequently, the argument that financial development might not have a monotonic impact on carbon emission intensity is further supported by the fact that inclusive financial growth increases carbon emission intensity. However, carbon emissions intensity decreases after a certain threshold level of financial development. The nonlinear nexus between inclusive financial development and environmental quality is weak in standalone countries. Overall, empirical results validate the existence of an inverted-U relationship between CO2 emissions and financial inclusion, indicating financial inclusion as a significant and positive driver in exerting environmental sustainability, consistent with [54] (2022) and [24].

Moreover, the nonlinear nexus between financial inclusion and carbon emission were also examined across different regions. The results in Table 12, Table 13, Table 14 reveal that financial inclusion-based EKC exists for Europe, the Middle East, Africa and Asia, whereas it does not hold for America. The presence of EKC in our results suggests a nonlinear relationship between CO2 emissions and inclusive financial development. The inconsistent behaviour of financial inclusion based on EKC is in line with the theoretical assumptions that ESG-related regulations and incentives for businesses and individuals to promote green growth varies across regions [20]. Hence, a policy that fits everyone is not an excellent way to ensure sustainable development [17].

**Table 12:** The Nonlinear relationship between Financial Inclusion and Carbon Emissions (Europe, Middle East and Africa region).

	D-K			GLM			Prais-Winsten		
Z_FI	0.305***			0.305***			0.283***		
	(0.0144)			(0.0173)			(0.0338)		
Z_FI2	0.0729***			0.0729***			0.0698***		
	(0.00628)			(0.00630)			(0.0108)		
Soft_FI		0.278***			0.278***			0.261***	
		(0.0123)			(0.0155)			(0.0302)	
Soft_FI2		0.0770***			0.0770***			0.0874***	
								
mmx_FI			0.304***			0.304***			0.230***
			(0.0232)			(0.0169)			(0.0296)
mmx_FI2			−0.0785***			−0.0785***			−0.0580***
			(0.00682)			(0.00664)			(0.00967)
Popg	−0.0101	−0.0113	−0.0120	−0.0101	−0.0113	−0.0120	−0.00855	−0.00860	−0.00802
	(0.0153)	(0.0148)	(0.0152)	(0.0112)	(0.0111)	(0.0111)	(0.00597)	(0.00598)	(0.00606)
l_gdp	0.230***	0.209**	0.0509	0.230***	0.209***	0.0509	0.116	0.124	0.212**
	(0.0785)	(0.0776)	(0.0414)	(0.0749)	(0.0741)	(0.0755)	(0.0875)	(0.0863)	(0.0890)
l_gdp2	−0.278*	−0.236	0.212**	−0.278**	−0.236*	0.212	0.376**	0.395**	0.578***
	(0.144)	(0.145)	(0.0844)	(0.131)	(0.129)	(0.130)	(0.160)	(0.157)	(0.161)
IND	0.0525***	0.0521***	0.0531***	0.0525***	0.0521***	0.0531***	0.0334***	0.0340***	0.0333***
	(0.00159)	(0.00150)	(0.00155)	(0.00206)	(0.00208)	(0.00206)	(0.00357)	(0.00352)	(0.00357)
TO	0.00118***	0.00122***	0.00129***	0.00118**	0.00122***	0.00129***	0.00285***	0.00276***	0.00357***
	(0.000214)	(0.000243)	(0.000262)	(0.000470)	(0.000462)	(0.000464)	(0.000856)	(0.000833)	(0.000839)
Hc	0.590***	0.586***	0.581***	0.590***	0.586***	0.581***	0.123**	0.131**	0.136**
	(0.0272)	(0.0300)	(0.0267)	(0.0433)	(0.0428)	(0.0432)	(0.0527)	(0.0520)	(0.0531)
Constant	−7.431***	−6.856***	−0.0883	−7.431***	−6.856***	−0.0883	3.354	3.576	5.731**
	(2.016)	(1.997)	(1.048)	(1.967)	(1.947)	(1.990)	(2.274)	(2.246)	(2.320)
R-squared	0.748	0.754	0.751				0.632	0.649	0.630
F-Stats (P Value)	0.000	0.000	0.000				0.000	0.000	0.000
Time Fixed Effects	Yes	Yes	Yes	Yes	Yes	Yes	Yes	Yes	Yes
Observations	850	850	850	850	850	850	850	850	850
No of Countries	50	50	50	50	50	50	50	50	50

**Table 13:** The Nonlinear relationship between Financial Inclusion and Carbon Emissions (Asia Region).

	D-K			GLM			Prais-Winsten		
Z_FI	0.483***			0.483***			0.537***		
	(0.0345)			(0.0337)			(0.0492)		
Z_FI2	−0.0693***			−0.0693***			−0.0680***		
	(0.0113)			(0.0138)			(0.0261)		
Soft_FI		0.443***			0.443***			0.493***	
		(0.0325)			(0.0296)			(0.0449)	
Soft_FI2		−0.0428***			−0.0428***			−0.0448	
		(0.0114)			(0.0151)			(0.0277)	
mmx_FI			0.468***			0.468***			0.465***
			(0.0268)			(0.0344)			(0.0480)
mmx_FI2			−0.0589***			−0.0589***			−0.0525**
			(0.0112)			(0.0123)			(0.0212)
Popg	0.319***	0.325***	0.330***	0.319***	0.325***	0.330***	0.0315	0.0310	0.0379
	(0.0631)	(0.0621)	(0.0619)	(0.0427)	(0.0416)	(0.0439)	(0.0244)	(0.0244)	(0.0257)
l_gdp	0.0969	0.125*	0.625***	0.0969	0.125	0.625***	0.128	0.140	0.392**
	(0.0626)	(0.0641)	(0.0797)	(0.127)	(0.126)	(0.138)	(0.156)	(0.156)	(0.164)
l_gdp2	0.441***	0.504***	1.279***	0.441**	0.504**	1.279***	0.447	0.483*	0.894***
	(0.138)	(0.149)	(0.128)	(0.215)	(0.213)	(0.227)	(0.275)	(0.274)	(0.284)
IND	0.0117**	0.0145***	0.0148***	0.0117***	0.0145***	0.0148***	0.00464	0.00619	0.00907
	(0.00477)	(0.00470)	(0.00443)	(0.00451)	(0.00449)	(0.00446)	(0.00810)	(0.00808)	(0.00835)
TO	0.000784**	0.000825*	0.000943**	0.000784**	0.000825**	0.000943**	0.00137*	0.00131*	0.00186**
	(0.000369)	(0.000402)	(0.000405)	(0.000398)	(0.000416)	(0.000399)	(0.000744)	(0.000760)	(0.000746)
Hc	0.517***	0.503***	0.558***	0.517***	0.503***	0.558***	0.162*	0.166*	0.244***
	(0.0432)	(0.0403)	(0.0465)	(0.0896)	(0.0870)	(0.0888)	(0.0862)	(0.0861)	(0.0881)
Constant	1.440	2.038	14.98***	1.440	2.038	14.98***	3.774	3.955	10.17**
	(1.601)	(1.615)	(2.210)	(3.336)	(3.301)	(3.679)	(4.112)	(4.094)	(4.351)
R-squared	0.918	0.920	0.912				0.715	0.719	0.691
F-Stats (P Value)	0.000	0.000	0.000				0.000	0.000	0.000
Time Fixed Effects	Yes	Yes	Yes	Yes	Yes	Yes	Yes	Yes	Yes
Observations	221	221	221	221	221	221	221	221	221
Number of groups	13	13	13	13	13	13	13	13	13

**Table 14:** The Nonlinear relationship between Financial Inclusion and Carbon Emissions (Americas Region).

	D-K			GLM			Prais-Winsten		
Z_FI	0.0862***			0.0862***			0.0928		
	(0.0166)			(0.0328)			(0.0716)		
Z_FI2	0.0482***			0.0482***			0.0639**		
	(0.0124)			(0.0137)			(0.0273)		
Soft_FI		0.0635***			0.0635*			0.118*	
		(0.0152)			(0.0348)			(0.0700)	
Soft_FI2		0.0464***			0.0464***			0.0767**	
		(0.0121)			(0.0155)			(0.0300)	
mmx_FI			0.0628**			0.0628*			0.115*
			(0.0250)			(0.0361)			(0.0670)
mmx_FI2			0.0467***			0.0467***			0.0548*
			(0.0148)			(0.0171)			(0.0303)
Popg	−0.298**	−0.299**	−0.327***	−0.298***	−0.299***	−0.327***	−0.0172	−0.0150	−0.0181
	(0.109)	(0.108)	(0.0993)	(0.102)	(0.102)	(0.104)	(0.0760)	(0.0765)	(0.0762)
l_gdp	0.162**	0.158**	0.0316	0.162	0.158	0.0316	0.180	0.191	0.197
	(0.0729)	(0.0738)	(0.0939)	(0.121)	(0.122)	(0.130)	(0.176)	(0.175)	(0.180)
l_gdp2	0.627***	0.617***	0.406**	0.627***	0.617***	0.406*	0.523*	0.545*	0.543*
	(0.146)	(0.146)	(0.177)	(0.197)	(0.198)	(0.207)	(0.315)	(0.312)	(0.316)
IND	−0.0135**	−0.0133**	−0.0139**	−0.0135**	−0.0133**	−0.0139**	−0.0194*	−0.0188*	−0.0188*
	(0.00469)	(0.00487)	(0.00501)	(0.00549)	(0.00557)	(0.00574)	(0.00990)	(0.00987)	(0.0105)
TO	0.00984***	0.00987***	0.00980***	0.00984***	0.00987***	0.00980***	0.00416*	0.00434*	0.00414*
	(0.000465)	(0.000445)	(0.000326)	(0.00149)	(0.00150)	(0.00153)	(0.00239)	(0.00239)	(0.00236)
Hc	1.131***	1.134***	1.131***	1.131***	1.134***	1.131***	0.260**	0.266**	0.253**
	(0.0892)	(0.0895)	(0.0981)	(0.113)	(0.116)	(0.117)	(0.122)	(0.122)	(0.120)
Constant	2.043	1.939	−1.270	2.043	1.939	−1.270	5.266	5.509	5.762
	(2.023)	(2.045)	(2.563)	(3.197)	(3.217)	(3.431)	(4.563)	(4.538)	(4.646)
Observations	117	117	117	117	117	117	117	117	117
R-squared	0.812	0.809	0.802				0.522	0.528	0.498
F-Stats (P Value)	0.000	0.000	0.000				0.000	0.000	0.000
Time Fixed Effects	Yes	Yes	Yes	Yes	Yes	Yes	Yes	Yes	Yes
Observations	187	187	187	187	187	187	187	187	187
Number of groups	11	11	11	11	11	11	11	11	11

Furthermore, by following the reasoning of [8], we also test the significance of inclusive financial system-based EKC in the presence of regular EKC across whole and subsamples, thereby incorporating the square term of GDP in Eq. (5). Our findings across whole and subsamples suggest the presence of EKC, and these results are robust and statistically significant for both the coefficients and squared terms of financial inclusion. It further shows that the financial inclusion-based EKC is independent of the regular EKC.

Regarding the region-based analysis, the long-term impact of financial inclusion on carbon emissions in Africa, Asia, Europe, and the Middle East is negative and promotes environmental sustainability. These regions have focused on alternate energy sources, including renewable energy, which allows for the necessary level of financial development that reduces carbon emissions. Several studies confirm the existence of the EKC inverted U-shaped hypothesis and the nonlinear negative impact of squared financial development on carbon dioxide emissions in different regions, including Central Asia and Europe [42], South Africa [55], Chine [56], UAE [57], on the other hand, demonstrates that none of the financial development indicators had a significant nonlinear influence on CO2 emissions in 46 sub-Saharan African countries. A linear relationship was observed between financial inclusion and carbon emissions in OECD and Asian countries [7,26]. According to these studies, financial development makes it easier to acquire loans for starting firms with high energy use, which adds to environmental degradation. In addition, financial growth also lowers transaction costs and makes lending to the private sector substantially more affordable. As a result, new projects are initiated without considering their environmental effects, which pollutes the environment.

### 4.4 Robustness check

Robustness tests are applied to verify the reliability of our inverted U-shaped relationship between carbon emission and financial inclusion. We employed GLM and Praise-Winsten panel data framework for full and subsamples. The GLM and Praise-Winsten test are consistent with our established results that inverted U-shaped relationships exist in developed, emerging and frontier markets except in standalone economies. Concerning the testing of region-wise nonlinearity nexus between the inclusive financial system and CO2 emissions, the results further validate our previous findings that the impact of financial inclusion-based EKC varies across regions.

Overall, the findings support the notion that the inverted U-shaped EKC hypothesis holds in the long run. The results support the notion that an inclusive financial system contributes to environmental degradation beyond inevitable financial development. Moreover, the non-persistent behaviour of financial inclusion-based EKC across the level of financial development and regions is consistent with the notion that the nonlinearity between financial sector development and carbon is conditioned upon solid governance mechanisms and strict environmental regulations [8]. The findings of this study shared common grounds with empirical studies such as [8,17], who have supported the nonlinear impact of financial inclusion on CO2 emissions.

## 5 Conclusion and policy implications

The current study investigated the empirical nexus between the financial inclusion and carbon emissions of full and heterogeneous subsample countries based on the EKC hypothesis. We analyse this relationship across different regions in view of STIRPAT framework. Our results from the D-K test illustrate that the inverted U-shaped relationship exists between CO2 and inclusive financial system in the entire sample and across heterogeneous subsamples. For the robustness check, we used GLM and Praise-Winsten panel data framework. Our results are consistent with our established results that inverted U-shaped relationships exist in developed, emerging and frontier markets except for standalone economies. Furthermore, the region-wise analysis also suggests the non-persistent behaviour of financial inclusion-based EKC across regions. We find inverted U-shaped relationships in Europe, the Middle East, Africa, and Asia. Further, we also document that this relationship does not exist in America.

We have derived several critical policy implications from the results. *First*, to attract private investment for low-carbon projects, financial inclusion can be used as a prerequisite. As financial services leverage private investment, that first needs the establishment of financial infrastructure. Ref. [58] documented that financial institutions are essential for innovative ideas such as the spillover effect of taxes and green credit guarantee schemes (GCGSs) that reduces the risk and attract private investors. The government should widen the financial coverage provided by the credit market, which led to the emergence of financial infrastructure by attracting financial institutions to meet the increased demand for financial services. *Second*, due to the heterogeneous response of financial inclusion in curtailing environmental degradation, governments should set a regulatory framework that promotes a reliable and inclusive financial system. In addition, consumers should be encouraged to enhance their financial literacy to best use the available financial services. *Third*, policy makers and regulators should identify issues related to financial regulation, inclusion and development as it is pertinent to implement policies that could reduce carbon emissions. Thus, a comprehensive green financing program should be developed by each country to achieve the 2030 SDGs of carbon neutrality and environmental sustainability. Additionally, authorities in these economies should adopt mitigating methods such as adopting and implementing digital financial inclusion in the future.

Besides, the study has a few limitations. *First*, this study covered 74 countries, regions, and subsamples to examine the nexus between financial inclusion and carbon emissions. Therefore, future studies could consider analysis based on a single country because these countries' economic, political, ethnic, cultural, and religious attributes vary. *Second*, the focus of this study remains solely on the relationship between financial inclusion and carbon emissions. However, other variables, such as the level of technological innovation and governance system of sample countries, may also affect environmental sustainability. Therefore, future studies may examine the moderating role of governance and technological innovation on the relationship between financial inclusion and environmental degradation. *Third*, to broaden policymakers' understanding, future researchers could use a more extensive set of financial inclusion proxies based on penetration, availability, and usage of the financial system to construct the financial inclusion index and check the relationship with carbon emissions using the same analytical framework.

### Author contribution statement

Shahzad Hussain: Conceived and designed the experiments.

Razia Gull: Performed the experiments.

Sabeeh Ullah: Contributed reagents, materials, analysis tools or data.

Abdul Waheed: Analysed and interpreted the data.

Muhammad Naeem: Contributed reagents, materials, analysis tools or data; Wrote the paper.

## Funding statement

This research did not receive any specific grant from funding agencies in the public, commercial, or not-for-profit sectors.

### Data availability statement

The data is Publicly available on world bank website.

## Declaration of interest's statement

The authors declare that they have no known competing financial interests or personal relationships that could have appeared to influence the work reported in this paper.

## Figures and Tables

**Table:** 

**Developed Markets**	**Emerging Markets**	**Frontier Markets**
Australia	Brazil	Bahrain
Austria	Chile	Bangladesh
Belgium	China	Croatia
Canada	Colombia	Estonia
Denmark	Czech Republic	Iceland
Finland	Greece	Jordan
France	Hungary	Kazakhstan
Germany	India	Kenya
Ireland	Indonesia	Lithuania
Israel	Kuwait	Mauritius
Italy	Malaysia	Morocco
Japan	Mexico	Nigeria
Netherlands	Peru	Oman
New Zealand	Philippines	Pakistan
Norway	Poland	Romania
Portugal	Qatar	Serbia
Singapore	Russian Federation	Slovenia
Spain	Saudi Arabia	Sri Lanka
Sweden	South Africa	Tunisia
Switzerland	Thailand	
United Kingdom	Turkey	
United States	United Arab Emirates	
**MSCI Standalone Markets**		
Argentina		
Bosnia and Herzegovina		
Botswana		
Bulgaria		
Jamaica		
Lebanon		
Malta		
Panama		
Trinidad and Tobago		
Ukraine		
Zimbabwe		
Region-Wise Division		
**Europe, Middle East & Africa**		**Asia**
Austria	Lithuania	Australia
Bahrain	Malta	Bangladesh
Belgium	Mauritius	China
Bosnia and Herzegovina	Morocco	India
Botswana	Netherlands	Indonesia
Bulgaria	Nigeria	Japan
Croatia	Norway	Malaysia
Czech Republic	Oman	New Zealand
Denmark	Poland	Pakistan
Estonia	Portugal	Philippines
Finland	Qatar	Singapore
France	Romania	Sri Lanka
Germany	Russian Federation	Thailand
Greece	Saudi Arabia	**Americas**
Hungary	Serbia	Argentina
Iceland	Slovenia	Brazil
Ireland	South Africa	Canada
Israel	Spain	Chile
Italy	Sweden	Colombia
Jordan	Switzerland	Jamaica
Kazakhstan	Tunisia	Mexico
Kenya	Turkey	Panama
Kuwait	Ukraine	Peru
Lebanon	United Arab Emirates	Trinidad and Tobago
	United Kingdom	United States
	Zimbabwe	

## Appendix

Table A1. Sample Description.

## Footnotes

1 https://ukcop26.org/.

2 https://unfccc.int/process-and-meetings/the-paris-agreement/the-paris-agreement#:∼:text=The%20Paris%20Agreement%20is%20a,compared%20to%20pre%2Dindustrial%20levels.

3 https://bills.parliament.uk/bills/3341.

4 https://sdgs.un.org/goals/goal13.

5 See https://www.statista.com/statistics/205966/world-carbon-dioxide-emissions-by-region/#:~:text=China%20alone%20accounted%20for%20nearly,tons%20of%20CO%E2%82%82%20were%20emitted.

6 World Health Organization. “WHO Director-General's Opening Remarks at the Media WBriefing on COVID-19–11 March 2020."
